# Assessing the impact of suppressing Southern Ocean SST variability in a coupled climate model

**DOI:** 10.1038/s41598-021-01306-2

**Published:** 2021-11-11

**Authors:** Ariaan Purich, Ghyslaine Boschat, Giovanni Liguori

**Affiliations:** 1 ARC Centre of Excellence for Climate Extremes, Sydney, Australia; 2 Climate Change Research Centre, University of New South Wales, Sydney, NSW Australia; 3 Bureau of Meteorology, Melbourne, VIC Australia; 4 School of Earth, Atmosphere and Environment, Monash University, Melbourne, VIC Australia; 5 Department of Physics and Astronomy, University of Bologna, Bologna, Italy

**Keywords:** Atmospheric dynamics, Physical oceanography, Climate and Earth system modelling

## Abstract

The Southern Ocean exerts a strong influence on global climate, regulating the storage and transport of heat, freshwater and carbon throughout the world’s oceans. While the majority of previous studies focus on how wind changes influence Southern Ocean circulation patterns, here we set out to explore potential feedbacks from the ocean to the atmosphere. To isolate the role of oceanic variability on Southern Hemisphere climate, we perform coupled climate model experiments in which Southern Ocean variability is suppressed by restoring sea surface temperatures (SST) over 40°–65°S to the model’s monthly mean climatology. We find that suppressing Southern Ocean SST variability does not impact the Southern Annular Mode, suggesting air–sea feedbacks do not play an important role in the persistence of the Southern Annular Mode in our model. Suppressing Southern Ocean SST variability does lead to robust mean-state changes in SST and sea ice. Changes in mixed layer processes and convection associated with the SST restoring lead to SST warming and a sea ice decline in southern high latitudes, and SST cooling in midlatitudes. These results highlight the impact non-linear processes can have on a model’s mean state, and the need to consider these when performing simulations of the Southern Ocean.

## Introduction

The Southern Ocean plays an important role in the global meridional overturning circulation and thus in the latitudinal transport of heat across the globe^1^. By dominating the net ocean heat^2^ and carbon^3^ uptake the Southern Ocean also plays a crucial role in the mitigation of ongoing global warming^4,5^. However, we are still unsure of the Southern Ocean's role in generating and/or modulating interannual to decadal atmospheric variability. To date most studies have focussed on the impact of atmospheric variability, such as variability associated with the Southern Annular Mode (SAM)^6,7^, zonal asymmetries linked with Zonal Wave 3^8^, and tropical-to-high latitude teleconnections^9,10^ on Southern Ocean and Antarctic sea-ice variability^11–22^. However, it is also plausible that sea surface temperature (SST) variability intrinsic to the Southern Ocean and/or resulting from the coupling between the ocean, sea ice and atmosphere, might play an important role in energizing or modulating atmospheric variability at interannual to decadal timescales^23^ with the longer timescales more likely to display such SST-driven variability^24^.

Compared to the atmosphere, the ocean is a slow-varying system and thus SST-driven atmospheric variability is potentially more predictable than purely intrinsic atmospheric variability. By comparing an atmosphere-only model with a fully coupled climate model, coupled air–sea processes were previously found to increase the atmospheric persistence of the SAM^25^. Furthermore, SST variability in the Southern Ocean may impact the latitudinal extent of Antarctic sea-ice, which has been shown to influence the latitude of the Southern Hemisphere mid‐latitude jet stream, although with some disagreement between studies based on different models: one study found increased Antarctic sea ice extent to cause a poleward shift in the jet, but no corresponding equatorward shift for reduced sea ice extent^26^; however in response to global warming conditions, various other studies have found reduced Antarctic sea ice extent to cause an equatorward shifted jet^27–30^.

Here, we use a fully coupled global climate model to investigate the role of Southern Ocean SST variability on the broader climate variability of the extratropical Southern Hemisphere. We perform a small ensemble in which the variability in the Southern Ocean over 40–65°S is suppressed by nudging the SST to the model monthly mean climatology. We find that the reduced SST variability causes robust mean-state changes in ocean and sea ice fields across the Southern Ocean; however, no clear influence is found on SAM variability, which appears to be internally generated. These results are in contrast with previous modelling studies^27–30^ suggesting that variability in sea-ice extent drives latitudinal shifts in the midlatitude Ferrel cell and influences the polarity of the SAM.

## Experiments

Using the Australian Community Climate and Earth System Simulator, version 1.0 (ACCESS1.0; see “Methods” section), we perform an ensemble of two Southern Ocean suppressed SST variability runs (identical except for their initial conditions), hereafter referred to as SOclimSST, and make use of three control runs (identical except their initial conditions), hereafter referred to as CTRL. All five runs are carried out over 1951–2001 with the same forcings as the ACCESS1.0 Coupled Model Intercomparison Project phase 5 (CMIP5) historical runs, including historically evolving atmospheric concentrations of greenhouse gases, aerosol emissions, and ozone. Restoring SST allows examination of the atmosphere and sea ice response to reduced SST variability. In the two SOclimSST runs, monthly-mean SST averaged over the three CTRL runs is restored over 40–65°S (with linear tapering 5° either side; Fig. S1 in the Supporting Information). The experiments have a restoring timescale of 2 days over the upper ocean model layer (10-m depth; equivalent to a 10-day timescale over 50-m depth). Commonly, a restoring timescale equivalent to a 30-day timescale over an upper ocean layer of 50-m depth is used when restoring SST in coupled models, which allows for large-scale thermal coupling of the ocean and atmosphere^31^. In ACCESS1.0 this would correspond to a restoring timescale of 6 days. The tighter timescale of 2 days applied in these experiments is chosen to strongly clamp down SST variability. All other model components (ocean, atmosphere and sea ice) evolve freely to this partial assimilation of the Southern Ocean. We show the influence of suppressing Southern Ocean SST variability by plotting the ensemble mean difference SOclimSST minus CTRL.

## Results

### Annual-mean response to reduced SST variability

**Figure 1:**
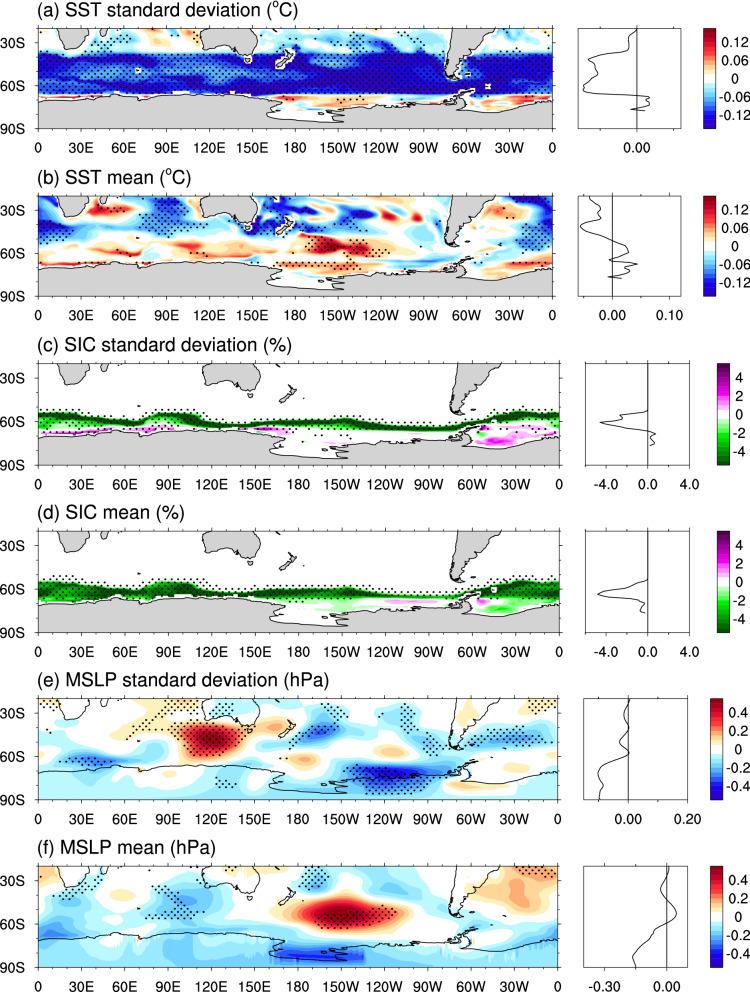
Annual-mean standard deviation and mean-state differences (SOclimSST minus CTRL) in: (**a**, **b**) SST, (**c**,**d**) SIC, and (**e**,**f**) MSLP. Stippling indicates robustness in the sign of the ensemble-mean difference, where the sign of all six individual differences (each SOclimSST run minus each CTRL run) is the same. Zonal mean panels are shown to the right of map panels. Figure produced using the NCAR Command Language (https://doi.org/10.5065/D6WD3XH5).

While the reduction of SST variability in the Southern Ocean (Fig. 1a) is a direct result of our experimental set-up, changes in mean-state SST, as well as in the mean state and variability of sea-ice concentration (SIC) and mean sea-level pressure (MSLP) are more difficult to interpret. The impact of SST restoring is largely limited to mid and high latitudes in the Southern Hemisphere, with little or no change in the variability of the Northern Hemisphere between the CTRL and SOclimSST runs (see Fig. S1 with global maps of SST standard deviation). The lack of full two-way air–sea coupling in the SOclimSST experiments results in a zonal-like response with a weak SST warming at high latitudes and a modest SST cooling at mid latitudes (Fig. 1b). This is an unexpected result, given that we are restoring SST with a tight restoring timescale, as discussed in more detail below. The outer sea ice zone lies within the SST restoring region, and in response to the reduced SST variability (Fig. 1a), there is also a robust (see “Methods”) reduction in SIC variability (Fig. 1c) in the outer ice zone. In contrast, a robust increase in SIC variability in some regions adjacent to the Antarctic coast (Fig. 1c) is also seen, discussed further below. The high-latitude SST warming is also associated with a robust mean-state reduction in circumpolar SIC (Fig. 1d).

In response to the reduced Southern Ocean SST variability, and the changed SST and SIC mean-state, changes in atmospheric circulation were anticipated^25,27–30^. However, changes in both MSLP variability and mean-state lack robustness in our experiments (Fig. 1e,f). Reduced MSLP variability and mean-state are seen across Antarctica and portions of the Southern Ocean (Fig. 1e,f; see also zonal-mean changes at right of panels). A localised region of increased MSLP variability is seen south of Australia, and increased mean-state MSLP is seen in the South Pacific, possibly a Rossby wave response to the SST cooling in the South Pacific Convergence Zone. Overall, however, MSLP changes are only robust in isolated patches. Given the small number of members in our ensembles (only two restoring runs and three control runs), the regionalised MSLP patterns shown in Fig. 1e,f are likely also due to intrinsic variability. A preliminary assessment of atmospheric changes in our experimental ensemble does not show robust changes in response to the SST restoring applied.

### Atmospheric response to reduced SST variability

**Figure 2:**
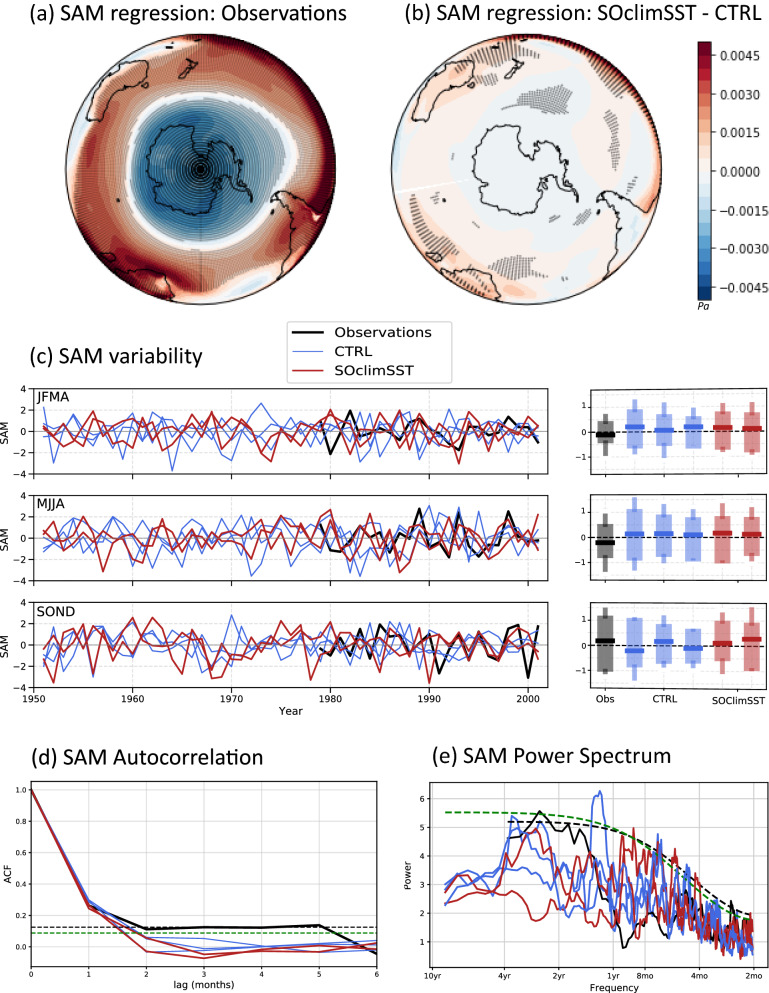
(**a**) Regression of monthly MSLP anomalies onto SAM in ERA5 reanalyses (1979–2001), and (**b**) difference between the regression of monthly MSLP anomalies onto SAM in SOclimSST runs and the regression of monthly MSLP anomalies onto SAM in CTRL runs (1951–2001). As in Fig. 1, stippling indicates robustness in the sign of the ensemble-mean difference, where the sign of all six individual differences (each SOclimSST run minus each CTRL run) is the same. (**c**) SAM timeseries for observations and each simulation for JFMA, MJJA and SOND, with corresponding bar plots on the right showing the median, 25th and 75th percentiles and ± one standard deviation for each timeseries. (**d**) Autocorrelation, and (**e**) power spectrum density in SAM across observations, CTRL and SOClimSST, with dashed lines showing the 95% confidence level for observations (black) and model runs (green). SAM has been calculated based on the Gong and Wang (1999) definition^35^ and all monthly data has been linearly detrended prior to analysis. Figure produced using Python (https://docs.python.org/3.0/).

From weekly to decadal timescales, the leading mode of atmospheric variability in the mid-to-high southern latitudes is the SAM^6,7^, with a positive SAM associated with a strengthening of the zonal atmospheric pressure gradient (see Fig. 2a) and a poleward shift in the jet and midlatitude storm tracks^32–34^. To further investigate changes to atmospheric circulation in our SOclimSST experiments, we assess changes in the SAM, defined here as the monthly difference in zonal MSLP between 40°S and 65°S^35^ (for comparison we also calculate an alternate SAM index^36^, shown in Fig. S2 in the Supporting Information). We detrend the monthly SAM time series over 1951–2001 and compare the SOclimSST and CTRL runs.

Overall, our experiments indicate that reducing SST variability in the Southern Ocean does not significantly impact the statistics of SAM in ACCESS1.0 (Fig. 2). The MSLP pattern typically associated with SAM in the SOclimSST experiment is of very similar structure to the SAM pattern in the CTRL runs on an annual timescale (Fig. 2b) albeit with a slight increase in MSLP over low latitudes. The interannual variability of the SAM index is explored in Fig. 2c, where timeseries are individually shown for each run, during late summer (JFMA), winter (MJJA) and spring (SOND; selection of 4-month seasons discussed below). SAM variations during the SOclimSST experiment (Fig. 2c red lines) typically remain within the range of variability of SAM in the CTRL runs (Fig. 2c blue lines) in all seasons, as also highlighted by the extent of the blue and red bar plots on the right-hand side of Fig. 2c. This range of variability includes observations, despite the short time record available for comparison here (see also Fig. S2 in the Supporting Information). There is no substantial change in the mean state or variability of SAM between the CTRL runs and SOClimSST experiment, further suggesting that the Southern Ocean does not play a dominant role in modulating the extratropical atmospheric circulation variability at these timescales in ACCESS1.0.

Autocorrelations of the SAM index (Fig. 2d) demonstrate a persistence that is marginally significant for 4–5 months in observations (although this becomes weak/insignificant when considering the longer station-based record^36^, see Fig. S2). While previous studies have shown that ACCESS1.0 tends to overestimate SAM persistence at time scales of less than a month^37^, for longer timescales the opposite occurs. Specifically, for lags larger than 1 month, the SAM persistence in ACCESS1.0 is essentially null, in contrast with the observations that shows a weak but consistently positive correlation up to 5-month lag. At lags larger than 1 month, the SAM’s autocorrelation reaches zero in all model runs, suggesting an equally weak persistence of the SAM in the CTRL runs and SOclimSST experiments. Reducing SST variability in the Southern Ocean has therefore not changed the temporal characteristics of SAM in ACCESS1.0, contrary to a previous study that found local air–sea coupling significantly increases the persistence of SAM over interseasonal to annual timescales^25^. This may also be due to the different approaches of our study to the previous study, which compared atmosphere only and coupled experiments to understand the influence of air–sea coupling^25^, whereas here we are comparing runs always within a coupled framework. However, the absence of difference between CTRL and SOclimSST experiments might simply reflect the inability of ACCESS1.0 to properly simulate the air–sea coupling mechanisms responsible for the observed longer memory of the SAM index. This may also highlight the need to use eddy-resolving models to better simulate air–sea feedbacks in key regions of the Southern Ocean^38–43^.

A complementary view of the characteristic timescale associated with the SAM index in both observations and simulations is obtained calculating the power spectrum density (Fig. 2e). Similar to the autocorrelation functions (Fig. 2d), there are no statistically significant differences among experiments, while the observations (black line, Fig. 2e) exhibit slightly increased variability in the range of 1–4 years. We note that the variations among CTRL runs suggest that the full extent of variability in the SAM may not be captured in these 51-year long runs. However, the few peaks above the red-noise significance levels in the runs are never consistent among members (CTRL or SOclimSST) and are thus determined to be the result of internal variability, and not due to the reduced SST variability in SOclimSST versus CTRL.

To further support this finding, we undertake maximum covariance analysis (MCA), which allows the extraction of the dominant co-varying patterns between two fields. We apply MCA to SST and MSLP monthly anomalies from the CTRL and SOclimSST runs individually, over 20–90°S. SST and MSLP patterns from the first mode, resembling the SAM, are shown in Fig. S3 in the Supporting Information. While the explained covariance is reduced in the SOclimSST runs, as expected given SST across the Southern Ocean is restored to the climatology, the MSLP patterns from all five runs are similar, strengthening our conclusion that in ACCESS1.0 reducing SST variability in the Southern Ocean does not affect the SAM. Overall, the significant but contained change (from ~ 55 to 40%) in the covariance explained by the leading MCA modes suggest that the ocean–atmosphere covariance is not strongly influenced by the coupling over the Southern Ocean. (We would, for example, expect a much larger difference in the explained covariance in restoring experiments targeting ENSO dynamics in the tropical Pacific).

### Ocean and sea ice responses to reduced SST variability

**Figure 3:**
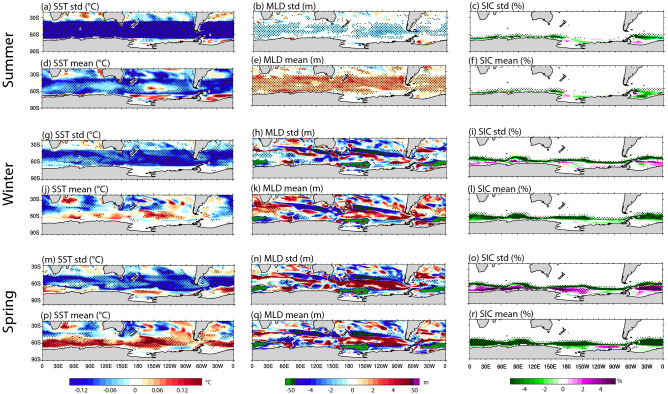
Seasonal standard deviation and mean-state differences (SOclimSST minus CTRL) for: (top two rows) late summer (JFMA), (middle two rows) winter (MJJA), and (bottom two rows) spring (SOND). (left columns) SST, (middle columns) MLD, (right columns) SIC. As in Fig. 1, stippling indicates robustness in the sign of the ensemble-mean difference, where the sign of all six individual differences (each SOclimSST run minus each CTRL run) is the same. Figure produced using the NCAR Command Language (https://doi.org/10.5065/D6WD3XH5).

To understand the mean-state ocean and sea ice changes discussed above, we consider the seasonal differences caused by reducing SST variability in our experiments. Here, we use three 4-month seasons, grouped by similar mean-state SST and mixed layer depth (MLD) differences in the restoring region: late summer (JFMA), associated with cool SST and deepened MLD (Fig. 3d,e); winter (MJJA), associated with weak, warm SST and strong but heterogenous MLD differences (Fig. 3j,k); and spring (SOND), associated with strong, warm SST and strong but heterogenous MLD differences (Fig. 3p,q). Overall, the SST difference patterns in winter and spring appear similar, but with the level of SST warming seen in spring much stronger than that seen in winter.

Mean-state SST changes under the strong SST restoring regime were not initially expected. In a coarse resolution ocean model simulation using the Modular Ocean Model (MOM; an early version of the ocean model component of ACCESS1.0), a marked reduction in stochastic variability from restoration resulted in nonlinear processes (e.g., mixed layer processes, ocean convection) that drove mean-state changes^44^. For example, over the high latitude Southern Ocean, an increase in ocean convection brings more subsurface heat to the relatively cooler surface. In our experiments, restoration restores surface temperature, but not surface buoyancy: SST restoration tries to offset surface warming from an increase in convection, which increases surface density, making the water column more unstable and further enhancing convection. Changes in the meridional overturning can also change the poleward transport of heat, influencing sea ice^44^.

In our experiments, reduced SST variability during late summer (Fig. 3a) is associated with a broad-scale reduction in MLD variability across the Southern Ocean (Fig. 3b). It is also associated with a broad-scale deepening of mean MLD (Fig. 3e), which is consistent with the mean SST cooling seen over the restoring region (Fig. 3d): during summer the ocean is receiving heat from the atmosphere, so a deeper mixed layer has more volume to heat, and thus is relatively cooler than a shallower mixed layer. We also note that the restoring regime leads to the strongest reduction in SST variability during late summer (Fig. 3a, cf. Fig. 3g, m) due to the climatologically highest variability during this season (not shown).

During winter and spring, the Southern Ocean mixed layer changes are highly spatially heterogeneous, although there is a general consensus between changes in variability and the mean state (regions of decreased variability tend to align with regions of mean-state decreases and vice versa) and patterns in winter are similar to spring. The Ross and Weddell Gyres show a slowdown of the spurious deep convection^45^ that occurs in these regions in response to the SST restoring applied (Fig. 3k,q; regions of green, noting the nonlinear colour bar). In response to the reduced deep convection, SST in the gyre regions actually increases in variability during spring, when climatological sea ice coverage is lower. Equatorward, in the South Pacific sector, there is a broad region of increased MLD variability and mean MLD, which corresponds to a region of strong SST warming: during winter the ocean is losing heat to the atmosphere, so a deeper mixed layer is consistent with a warmer surface. In other sectors, MLD and SST changes do not always align: while MLD changes are spatially heterogeneous around the Antarctic coast, by spring, warm SSTs are seen around the whole coastline. To summarise, across the SST restoring region (40–65°S), late summer SST cooling contrasts winter and spring SST warming (Fig. 3), with the latter dominating the annual mean difference (weak broad-scale warming across the Southern Ocean; Fig. 1b).

Sea ice changes largely reflect high-latitude SST changes in all seasons. Annual mean-state changes are driven by mean-state reductions in winter (Fig. 3l), and particularly in spring SIC (Fig. 3r), the latter which co-occurs with the strongest mean-state SST warming (Fig. 3p). During spring a small change in mean-state SST due to the restoring regime may be amplified by the positive sea ice-albedo feedback^46^ at high latitudes, leading to a stronger SST warming and sea ice decline. Mean-state SIC is also reduced during late summer (Fig. 3f), likely following the reduced spring mean-state SIC^46,47^. Interestingly, winter and spring SIC variability shows a reduction in the outer ice zone (Fig. 3i,o) but increased SIC variability in the inner ice zone, driving the inner-ice zone variability increase seen in the annual difference (Fig. 1c). This dipole response in variability is likely caused by the surface warming and mean-state reduction in SIC in both regions: in the outer ice zone, surface warming may act to remove ice in the region completely, thus reducing SIC variability. Conversely, in the inner ice zone, surface warming would allow a larger range of sea ice conditions (from completely ice covered to more ice-free regions) to occur, thus increasing SIC variability.

### Processes driving mean-state changes

**Figure 4:**
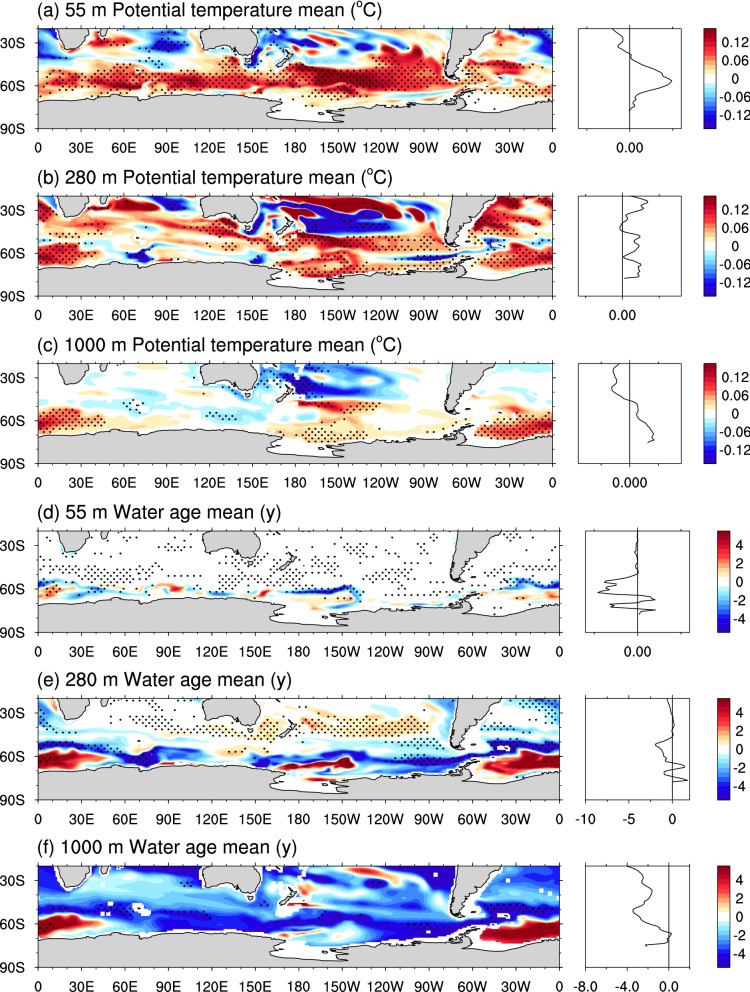
As for Fig. 1, but for annual-mean mean-state differences in: (**a**,**b**,**c**) potential temperature at 55 m, 280 m and 1000 m, respectively, and (**d**,**e**,**f**) water age at 55 m, 280 m and 1000 m, respectively. Figure produced using the NCAR Command Language (https://doi.org/10.5065/D6WD3XH5).

To gain insight into the processes driving the mean-state SST and sea ice changes, we assess mean-state differences in subsurface temperature and water age (which acts as a tracer in ACCESS1.0, indicating the length of time since a parcel of water has been at the surface^48^). A broad-scale annual-mean warming is seen across the Southern Ocean, particularly strong at 55-m depth (Fig. 4a), and reflecting the SST mean-state warming seen in the annual mean (Fig. 1b) and particularly during spring (SOND; Fig. 3p). The warming extends to 280-m depth in most regions (Fig. 4b), and 1000-m depth in the Ross and Weddell Gyres (Fig. 4c). Little change in water age is seen equatorward of the Antarctic margins at 55-m depth (Fig. 4d), as this depth is within the mixed layer; however, at depths of 280 m and 1000 m there is a broad scale decrease in water age (Fig. 4e,f). In the Ross and Weddell Gyres there is an increase in water age (Fig. 4e,f). Considered together, these potential temperature and water age difference patterns indicate a broad-scale increase in convective mixing across the Southern Ocean, particularly over the 50–60°S latitude band, which brings warm subsurface waters to the surface, leading to the broad-scale warming seen at 55 and 280-m depths (Fig. 4a, b) and mixes young surface waters into the subsurface, decreasing the water age at 280 m (Fig. 3e). Increased convective mixing is also consistent with the deeper MLD seen across this band (e.g. Fig. 3e,k,q, also Fig. S4). Changes in the Ross and Weddell Gyres are indicative of a shutdown of the deep convection that occurs in these regions (with less deep convection, water is less vented, so the time since it has been at the surface increases; Fig. 4e,f). These processes must occur quite vigorously on daily and/or sub-daily timescales, given the restoring timescale of 2 days in our simulations.

These changes in convective mixing are in agreement with non-linear mixed layer feedbacks in a previous study^44^. Changes in the meridional overturning circulation (MOC) can also occur in response to SST restoring, which may impact SST and sea ice^44^. Our SOclimSST experiments show a reduction in the global MOC over 40–55°S, and a weak increase in the MOC over 60–75°S, increasing the poleward heat transport (Fig. S4). These changes increase the poleward transport of heat, causing a high latitude warming and sea-ice decline, and a mid-latitude to tropical cooling, as seen in annual mean SST and 55 m potential temperature changes. Thus, MOC changes in response to the Southern Ocean SST restoring applied also likely contribute to the mean-state changes.

Our analysis suggests that oceanic changes are responsible for the mean-state SST and sea ice changes in our experiments, rather than atmospheric changes that then feedback to the ocean. Further support for the role of the ocean in driving these mean state changes comes from the relationship between zonal mean SST and total surface heat flux (Fig. S5), that suggests SST changes are driving heat flux changes, and not the other way around. During summer, SST warms over 65–70°S and the total heat flux into the ocean decreases: decreased heat flux into the ocean cannot drive an SST warming, but rather the warmer SST leads to a larger sensible and latent heat flux from the ocean to the atmosphere (as previously found for the opposite situation of cool SST and increased heat fluxes into the ocean^48^). During winter and spring, and further equatorward, decreased total surface heat fluxes into the ocean between 55 and 65°S again suggest that the surface heat fluxes largely respond to the SST and sea ice changes, not the other way around. In particular, the heat flux changes are an indicator of decreased sea ice and surface warming in response to the changes in restoring and ocean circulation (i.e., more heat is lost to the atmosphere when ice cover is decreased, and in ice-free areas more sensible heat is transferred from the warmer ocean surface to the atmosphere).

## Discussion

With the aim of understanding the role of oceanic variability on mid-to-high latitude Southern Hemisphere climate, we conduct partially coupled climate model simulations using ACCESS1.0, a global coupled climate model, in which Southern Ocean variability is restored to the model monthly mean climatology over 40–65°S. All other model components evolve freely to this partial assimilation of the Southern Ocean. We find that suppressing Southern Ocean SST variability does not impact the SAM in our simulations. This might suggest that air–sea feedbacks do not play an important role in the persistence of the SAM, however, based on the findings presented here, it more likely reflects the inability of ACCESS1.0 to properly simulate the air–sea coupling mechanisms responsible for the observed longer memory of the SAM index. The observed SAM index exhibits marginally significant autocorrelations for lags of 4–5 months, however in ACCESS1.0 at lags greater than 1 month, the SAM’s autocorrelation reaches zero (Fig. 2d). A number of studies have highlighted the importance of eddies over the Southern Ocean^43^ and have suggested that eddy-resolving models are required to better simulate air–sea feedbacks in the region^38–42^. Further, SST fronts influence the formation of atmospheric fronts^49^, and mid-latitude ocean fronts have been shown to amplify and anchor Southern Hemisphere baroclinic annular mode variability, which may in turn help maintain the SAM^50^. Resolving SST fronts with a high-resolution ocean component has been shown to influence air–sea fluxes^51^. Thus, to fully understand the impact of oceanic variability on Southern Hemisphere climate, further coupled experiments performed in an eddy-resolving framework are required.

By experimental design, SST restoring changes the SST variability, but it also changes convection and mixed layer processes in the model^44^, and these non-linear oceanic changes drive changes in SST and sea ice mean states that are robust (e.g., Fig. 1b, d). These changes occur despite the tight restoring timescale in our simulations. SST restoring leads to a broad scale (although spatially variable) increase in MLD across 50–60°S, but also a decrease in MLD associated with a shutdown of the deep convection in the Ross and Weddell Gyres. Associated with these changes, our runs show an annual-mean SST warming and sea ice decline at southern high latitudes, and SST cooling in midlatitudes. SST restoring also leads to a reduction in the upper branch of the southern MOC cell (Fig. S4), increasing the heat transport to the high latitudes and decreasing the heat transport to the mid latitudes, further contributing to the SST warming and cooling in these regions respectively.

These results highlight the impact that non-linear processes can have on a model’s mean state, and the need to consider these when performing simulations of the Southern Ocean. “Pacemaker” style runs with regional SST restored to a desired monthly value are now common place^52^ and associated with this experimental design and as emphasised in our study, the SST variability will also change. In such cases the magnitude of the difference between the freely running model and the SST to which the model is being restored to may be greater than the mean-state SST changes seen here (of order 0.1 °C). Nevertheless, the findings here demonstrate that in such experiments, analysis in and near the restoring region should be treated carefully in light of the non-linear feedbacks between the restoring, convection and mixed layer processes.

## Methods

Our experiments are run with ACCESS1.0, developed by the Commonwealth Scientific and Industrial Research Organisation and the Australian Bureau of Meteorology^53^*.* ACCESS1.0 is one of the best performing CMIP5 models at simulating regional Southern Hemisphere climate and Antarctic sea ice extent^54,55^. ACCESS1.0 has an atmosphere component (HadGEM2, r1.1) with N96 horizontal resolution and 38 vertical levels, and ocean (MOM4p1) and sea ice (CICE4.1) components with 360 longitude by 300 latitude points on a rectangular grid with enhanced resolution at the equator, and 50 vertical levels in the ocean^53^.

In our SOclimSST experiments, SST over 40–65°S is restored to the monthly-mean SST averaged over 1960–1990 from the average of three CTRL runs. We show the influence of suppressing Southern Ocean SST variability by plotting the ensemble mean difference SOclimSST minus CTRL over the full 1951–2001 period, however results are qualitatively very similar when analysed over 1960–1990, and conclusions remain the same.

We assess the robustness of the ensemble-mean difference by considering the sign of all six individual differences (each SOclimSST run minus each CTRL run; for example, at each grid point we consider the signs of the difference in SOclimSST run 1 minus CTRL run 1, SOclimSST run 1 minus CTRL run 2 and so on) and indicate robustness when the signs of all six individual differences are the same.

We compare our model results to observations from ERA5^56^ and NOAA ERSSTv5^57^. We also make use of the Marshall station-based SAM index^36^.

## Supplementary Information

Supplementary Figures.
