# Publisher Correction: Assessing the impact of suppressing Southern Ocean SST variability in a coupled climate model

**DOI:** 10.1038/s41598-021-02519-1

**Published:** 2021-11-23

**Authors:** Ariaan Purich, Ghyslaine Boschat, Giovanni Liguori

**Affiliations:** 1 ARC Centre of Excellence for Climate Extremes, Sydney, Australia; 2 Climate Change Research Centre, University of New South Wales, Sydney, NSW Australia; 3 Bureau of Meteorology, Melbourne, VIC Australia; 4 School of Earth, Atmosphere and Environment, Monash University, Melbourne, VIC Australia; 5 Department of Physics and Astronomy, University of Bologna, Bologna, Italy

Correction to: *Scientific Reports* https://doi.org/10.1038/s41598-021-01306-2, published online 11 November 2021

The original version of this Article contained an error in Figure 3 where all the panels displayed an additional diagonal line. This has now been removed. The original Figure 3 and accompanying legend appear below.

The original Article has been corrected.

**Figure 3:**
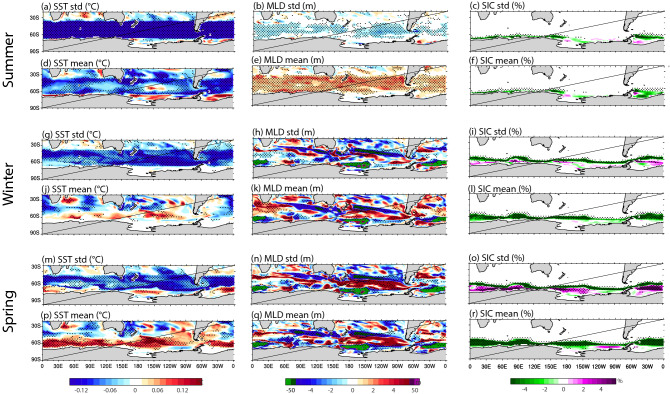
Seasonal standard deviation and mean-state differences (SOclimSST minus CTRL) for: (top two rows) late summer (JFMA), (middle two rows) winter (MJJA), and (bottom two rows) spring (SOND). (left columns) SST, (middle columns) MLD, (right columns) SIC. As in Fig. 1, stippling indicates robustness in the sign of the ensemble-mean difference, where the sign of all six individual differences (each SOclimSST run minus each CTRL run) is the same. Figure produced using the NCAR Command Language (https://doi.org/10.5065/D6WD3XH5).

