# Supplementary material for: Assessing the impact of suppressing Southern Ocean SST variability in a coupled climate model
Source: Sci Rep. 2021 Nov 11;11:22069. doi: 10.1038/s41598-021-01306-2 (PMC8585967; doi:10.1038/s41598-021-01306-2)
Supplement: Supplementary file 1 — Supplementary Figures. [file 41598_2021_1306_MOESM1_ESM.pdf]

Supplementary information for:

Assessing the impact of suppressing Southern Ocean SST variability in a coupled climate model

Ariaan Purich[*,1,2], Ghyslaine Boschat[1,3] and Giovanni Liguori [1,4,5]

[1] *ARC Centre of Excellence for Climate Extremes, Australia*

[2] *Climate Change Research Centre, University of New South Wales, Sydney NSW, Australia*

[3] *Bureau of Meteorology, Melbourne VIC, Australia*

[4] *School of Earth, Atmosphere and Environment, Monash University, Melbourne VIC, Australia*

[5] *Department of Physics and Astronomy, University of Bologna, Bologna, Italy*

[*] *Corresponding author: a.purich@unsw.edu.au*

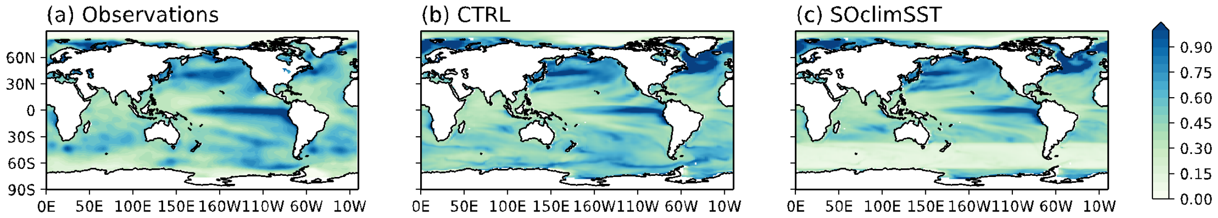

Figure S1: Standard deviation of monthly SST anomalies over 1951-2001 in: (a) observations (NOAA ERSSTv5), (b) CTRL, and (c) SOclimSST. The region of SST restoring and reduced SST variability, 40-65°S, can be seen in (c). Figure produced using Python (https://docs.python.org/3.0/).

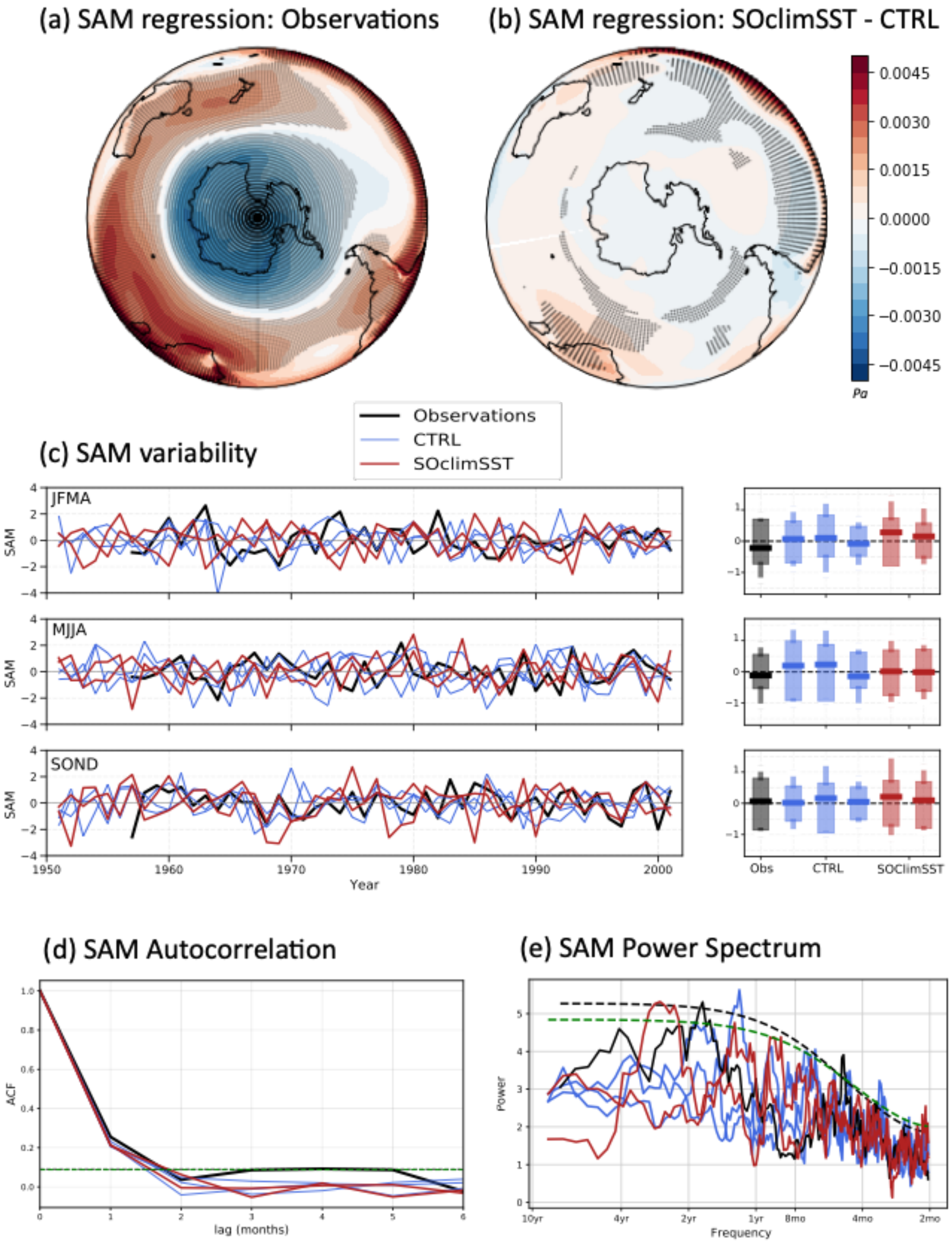

Figure S2: As for Figure 2 but for the SAM index calculated as in Marshall (2003). Observations over 1957-2001. Figure produced using Python (https://docs.python.org/3.0/).

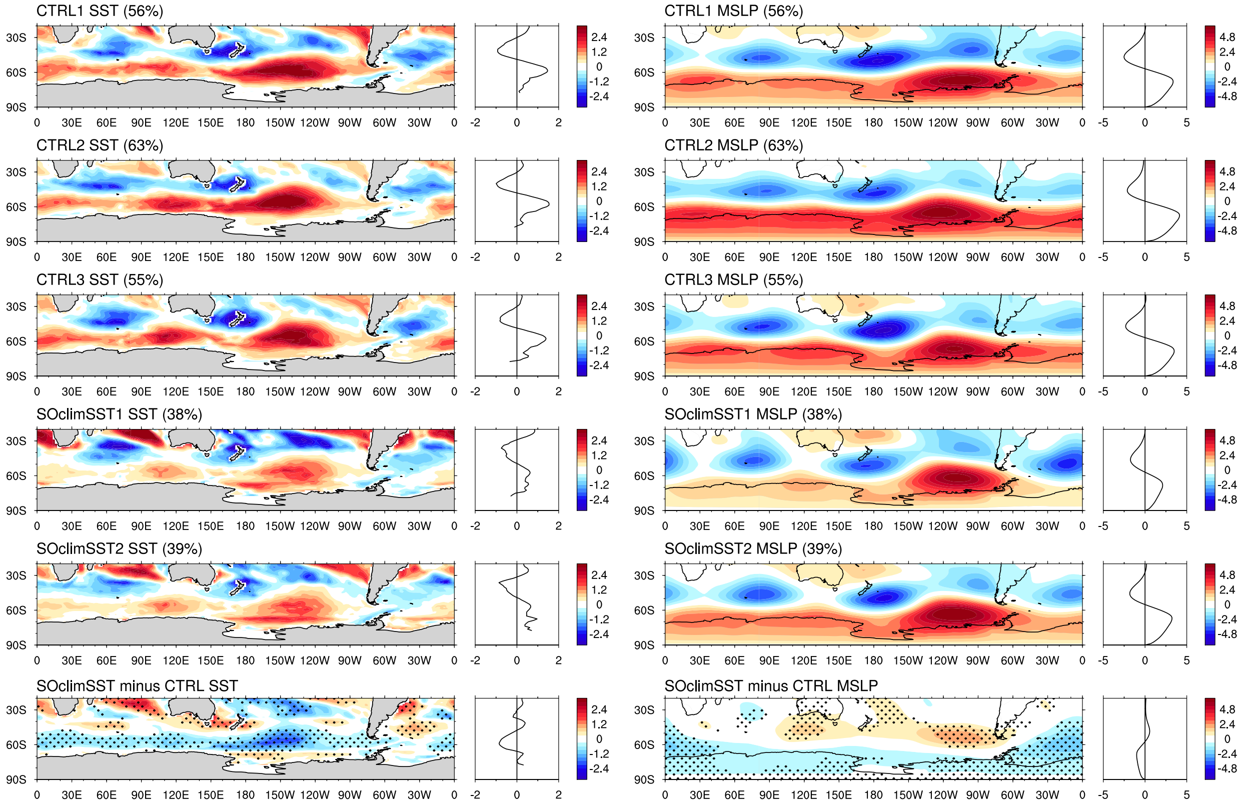

Figure S3: (Left) SST, and (right) MSLP patterns from the first mode of MCA, undertaken over 20-90°S, on each run individually. The explained variance for the first mode is indicated in brackets above each panel. (Bottom) ensemble-mean difference pattern for SOclimSST minus CTRL, with stippling indicating robustness as in Figure 1. Zonal mean panels are shown to the right of map panels. Figure produced using the NCAR Command Language (https://doi.org/10.5065/D6WD3XH5).

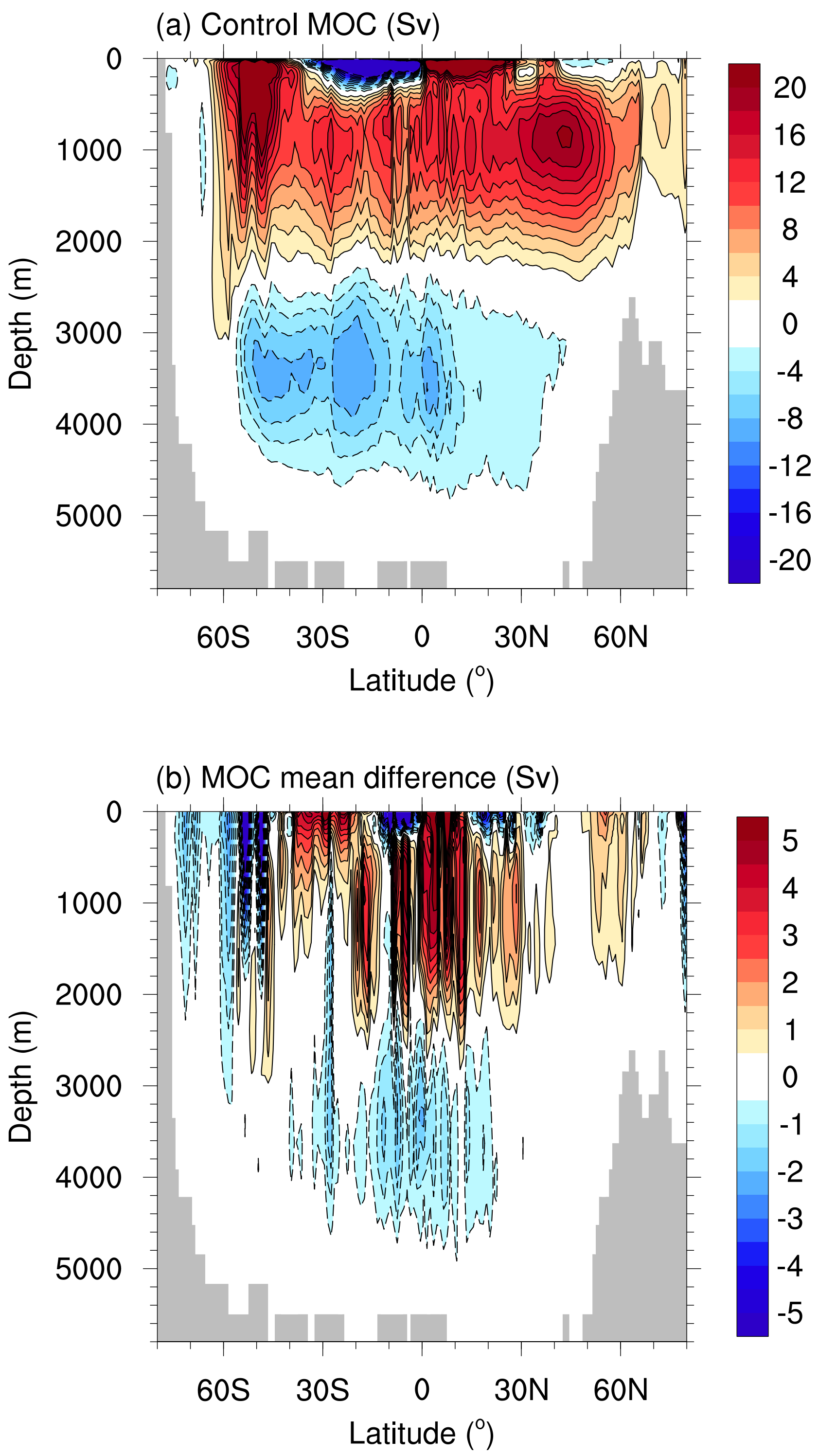

Figure S4: Global MOC: (a) CTRL mean-state, and (b) mean-state difference (SOclimSST minus CTRL, noting that only two CTRL runs are used here due to data availability). Figure produced using the NCAR Command Language (https://doi.org/10.5065/D6WD3XH5).

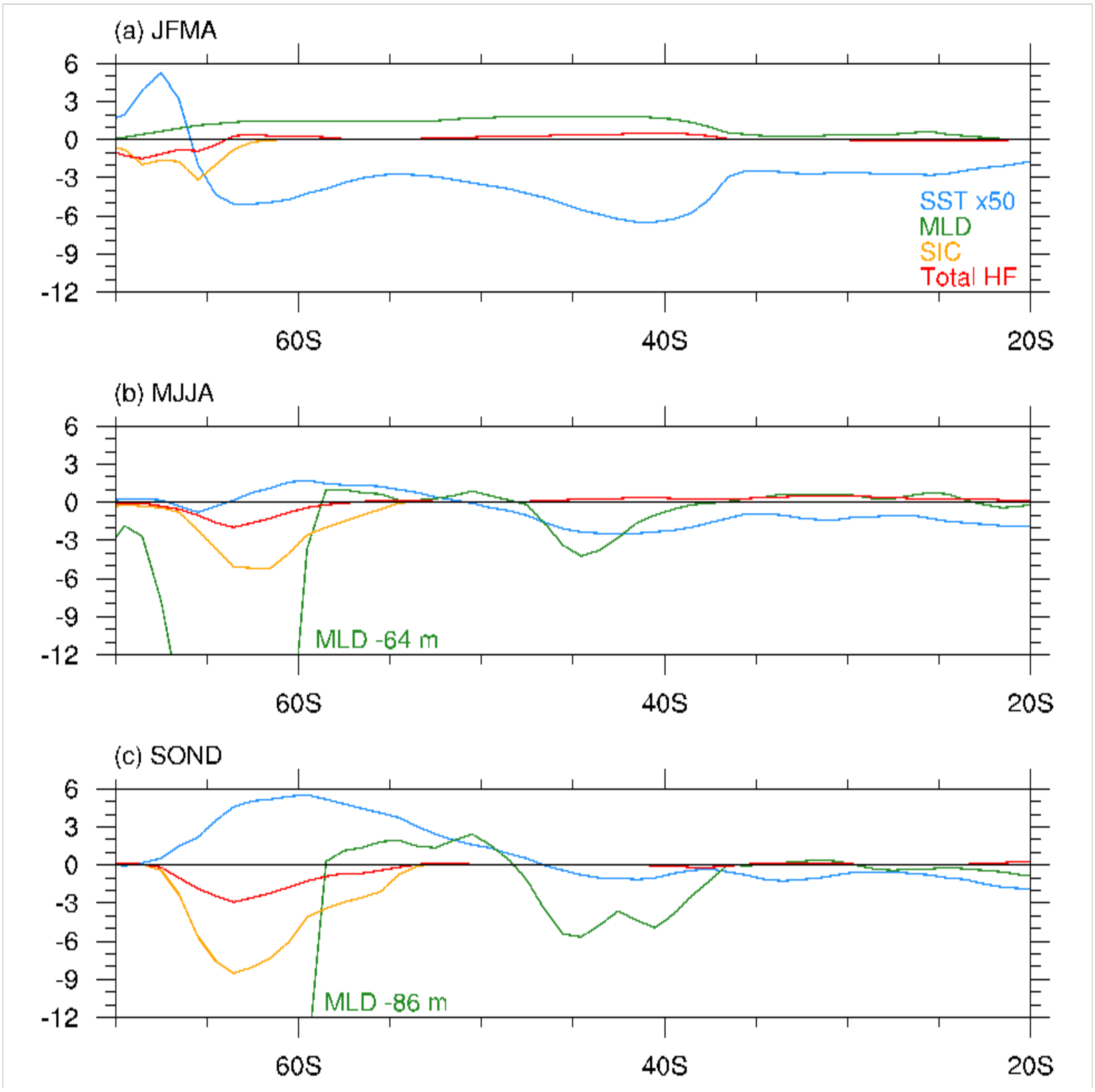

Figure S5: Ensemble-mean differences in zonal-mean SST (blue, multiplied by a scale factor of 50 to be shown on the same y-axis), MLD (green), SIC (yellow) and total downward surface heat fluxes (red) for: (a) austral late summer (JFMA), (b) austral winter (MJJA), and (c) austral spring (SOND). Total heat flux data is only available for one CTRL run. Figure produced using the NCAR Command Language (https://doi.org/10.5065/D6WD3XH5).
